# Vagotomy and the incidence of rheumatoid arthritis and osteoarthritis: a Danish register-based study

**DOI:** 10.1186/s13075-025-03567-y

**Published:** 2025-05-16

**Authors:** Matthew C. Baker, Dávid Nagy, Suzanne Tamang, Erzsébet Horváth-Puhó, Henrik Toft Sørensen

**Affiliations:** 1 Division of Immunology and Rheumatology, Department of Medicine, Stanford University, (MCB, ST), Stanford, CA USA; 2 Department of Clinical Epidemiology, Department of Clinical Medicine and Center for Population Medicine, Aarhus University Hospital, Aarhus University, (DN, EHP, HTS), Aarhus, Denmark; 3 Clinical Excellence Science Center, Stanford University, (HTS), Stanford, CA USA

**Keywords:** Vagus nerve, Rheumatoid arthritis, Osteoarthritis, Vagus nerve stimulation, Vagotomy, Rheumatoid arthritis risk

## Abstract

**Objectives:**

Given the potential role of vagus nerve stimulation in treating rheumatoid arthritis (RA), we examined the incidence of RA and osteoarthritis (OA) in patients who underwent different forms of vagotomy that disparately affect the inflammatory reflex.

**Methods:**

Using nationwide health registries, we constructed cohorts of patients in Denmark who underwent truncal or superselective vagotomy between 1977 and 1995 and comparison members from the general population matched 10:1 on birth year, sex, and calendar year. We identified incident RA or OA and used Cox proportional hazards models to compute adjusted hazard ratios (aHRs) and corresponding 95% CI.

**Results:**

Our cohorts consisted of 2,260 truncal vagotomy patients matched with 22,610 comparators, and 3,810 superselective vagotomy patients matched with 38,090 comparators. The incidence rate (IR) of RA per 1,000 person-years (95% CI) in the truncal vagotomy cohort was 10.2 (6.5–15.3) versus 7.2 (6.1–8.4) in the matched comparison cohort. The aHR (95% CI) for RA development was 2.62 (1.47–4.67) in the truncal vagotomy cohort and 1.05 (0.51–2.17) in the superselective vagotomy cohort, with respect to comparison cohorts. The risk of developing OA was not significantly different for either vagotomy cohort compared with comparison cohorts.

**Conclusion:**

Truncal vagotomy was associated with an increased incidence of RA; this association was not observed with superselective vagotomy. No association with either form of vagotomy was seen with OA. These findings support the hypothesis that disruption of vagus nerve signaling impacts the inflammatory reflex and contributes to the development of RA.

**Supplementary Information:**

The online version contains supplementary material available at 10.1186/s13075-025-03567-y.

## Introduction

Rheumatoid arthritis (RA), an autoimmune rheumatic disease characterized by joint inflammation and damage as well as systemic features, affects approximately 1% of the population worldwide [1, 2]. Vagus nerve stimulation (VNS) is being investigated as a potential RA therapy [3]. VNS is believed to function through the inflammatory reflex, which refers to a primitive connection between the nervous system and the immune system. Vagus nerve sensory afferents convey inflammatory signals to the brainstem, and efferent signals traverse the splenic nerve, ultimately leading to acetylcholine release in the spleen and inhibition of proinflammatory cytokine production by macrophages [4–8]. Prior studies of an implantable cervical vagus nerve stimulator have shown preliminary efficacy in patients with RA, whereas a larger, randomized, double-blind, sham-controlled study of an auricular vagus nerve stimulator did not demonstrate benefit [5, 9, 10]. The potential role of VNS in treating RA remains unclear.

The vagus nerve has been a target of various therapeutic interventions for many decades. Starting in the 1940s, vagotomy was introduced to treat refractory peptic ulcer disease (PUD) [11]. Over time, the methods for treating PUD with vagotomy evolved into three primary techniques: (1) full truncal vagotomy, in which two or more vagal trunks are divided as they enter the abdominal cavity, at or below the esophageal hiatus; (2) selective vagotomy, in which the hepatic branch of the left (anterior) vagus nerve and the celiac branch of the right (posterior) nerve are transected, thereby achieving total gastric denervation but leaving hepatic, biliary, and visceral vagal fibers intact; and (3) superselective vagotomy, in which only the direct vagal inputs to the stomach are affected [11, 12].

The aim of this study was to examine the incidence of RA or OA development after various vagotomy methods with potentially differing effects on the inflammatory reflex. We hypothesized that full truncal vagotomy would increase the risk of RA development, as interrupting vagus nerve signaling may result in increased inflammatory cytokine production by macrophages, and thus the development of RA in susceptible individuals. We hypothesized that full truncal vagotomy would not affect the risk of OA development, as OA pathogenesis is not primarily driven by inflammatory cytokines. Finally, we hypothesized that superselective vagotomy would not influence either condition, as this method has no effect on vagus nerve signaling to the spleen, and therefore the inflammatory reflex remains intact.

## Materials and methods

### Study design and data source

This population-based cohort study used prospectively collected data from Denmark, whose tax-supported health care system provides free care [13]. Data between January 1, 1977 and December 31, 2018 were obtained from the Danish Civil Registration System (CRS) and the Danish National Patient Registry (DNPR) [13].

The CRS was used to define date of birth, sex, date of death, and emigration status. The DNPR, containing data on nonpsychiatric hospitalizations since 1977 and outpatient clinic and emergency room visits since 1995, was used to find patients who had and had not undergone vagotomy, as well as their comorbid conditions.

### Study population

The exposed cohorts included individuals who underwent vagotomy according to Danish Classification of Surgical Procedure and Therapies Codes (Supplementary Table 1), were registered in the CRS at least two years before the date of vagotomy (the index date), and who did not have a prior RA or OA diagnoses at any time before the index date. Individuals in the exposed cohorts were further classified according to Danish Classification of Surgical Procedure and Therapies Codes into truncal vagotomy, selective vagotomy, and superselective vagotomy cohorts. Patients who underwent selective vagotomy were excluded, as residual vagus nerve signaling to the celiac plexus and splenic nerve might have confounded the results. In the comparison cohort, individuals who had not undergone vagotomy were randomly selected from the general population with replacement and matched 10:1 on birth year, sex, and calendar year [14]. The date of vagotomy for each vagotomy patient served as the index date for the matched comparison cohort. Patients who underwent full truncal vagotomy those who underwent superselective vagotomy were each compared with their matched comparators.

### Outcome ascertainment

The primary outcome was the incidence rate (IR) of RA or OA after the index date. Outcomes were identified in the DNPR according to a primary or secondary discharge diagnosis from an inpatient or outpatient clinic. Discharge diagnoses are recorded with the International Classification of Diseases (ICD) codes in the DNPR. Incident RA or OA was defined by the presence of two or more ICD-8 or ICD-10 codes separated by 7 to 365 days (Supplementary Table 2) [15]. Joint replacement was assessed according to a diagnosis of OA and a procedure code for knee or hip arthroplasty (Supplementary Table 3). Patients were followed from the index date until a diagnosis of RA or OA, death, emigration, 10 years of follow-up, or the end of the study (December 31, 2018).

### Covariables

Patient birth year, sex, and the calendar year of the index date were obtained from the CRS. The Charlson comorbidity index score was calculated on the basis of all diagnosis codes before the index date (list of relevant diagnosis codes in Supplementary Table 4).

### Statistical analysis

Patients who underwent truncal or superselective vagotomy were matched on birth year and sex 10:1 with people who had not undergone vagotomy. The truncal vagotomy and superselective vagotomy cohorts were each compared with matched general population comparison cohorts. The incidence of RA and OA was calculated with a cumulative incidence function, with death treated as a competing event, and the incidence rate per 1,000 person-years reported. Cox proportional hazards regression models were applied to compute hazard ratios (HRs) and corresponding 95% confidence interval (CIs), with adjustment for age at index date, sex, and calendar year of the index date. The proportional hazards assumption was assessed graphically with log-minus-log plots, and no major violations were detected. Similar analyses were conducted for the truncal vagotomy versus superselective vagotomy cohorts, which were unmatched cohorts.

Sensitivity analysis was performed with the Fine-Gray model, given the strong competing risk of death in the truncal vagotomy cohort [16].

Statistical analyses were conducted in SAS version 9.4 (SAS Institute Inc., Cary, NC, USA). The presented absolute numbers were rounded to the nearest ten, in compliance with Danish data protection rules. This study was reported to the Danish Data Protection Agency (record number 2016-051-000001/ 2234) by Aarhus University.

## Results

**Fig. 1:**
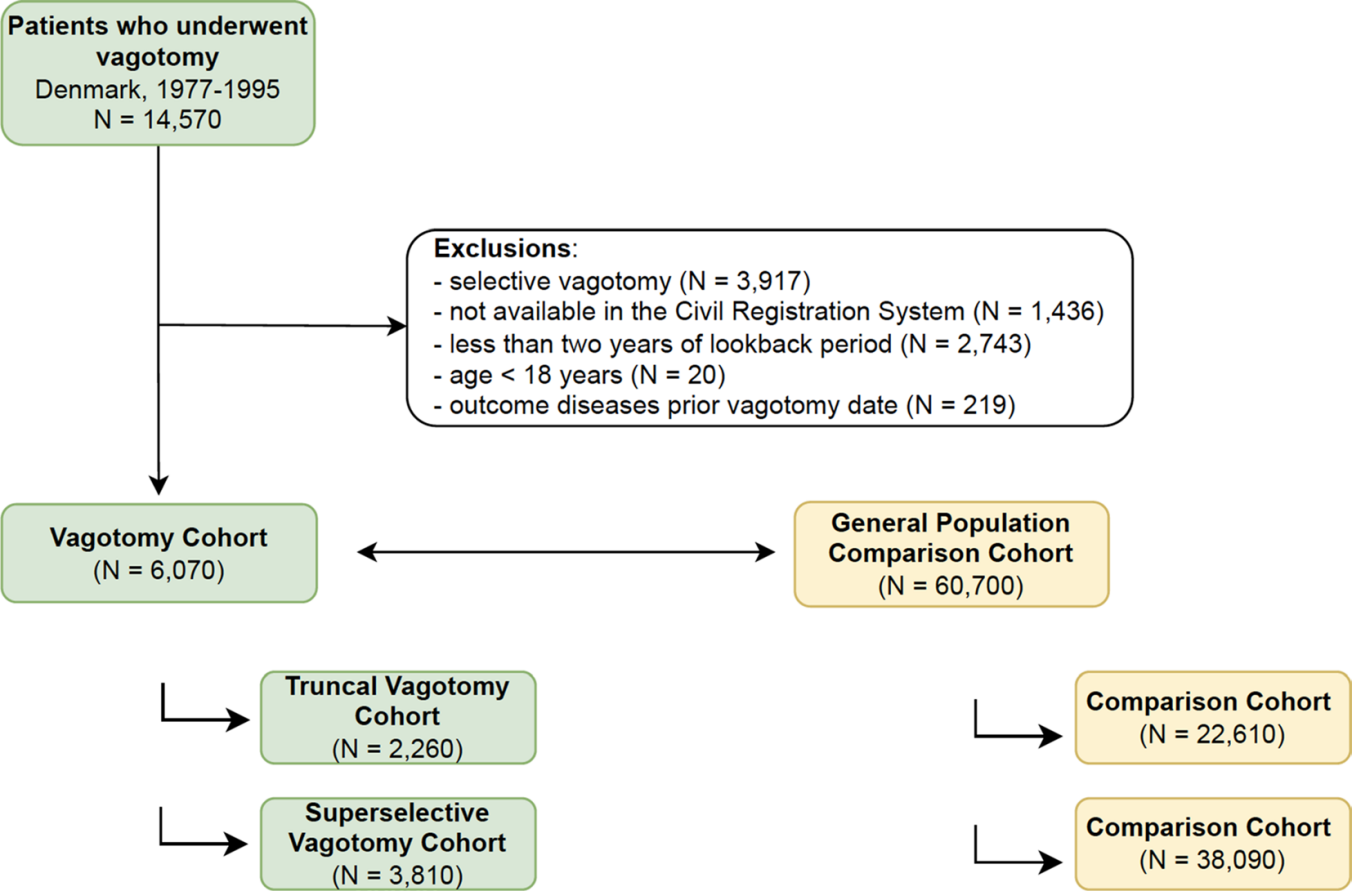
Cohort selection

We identified 2,260 patients who underwent truncal vagotomy, who were matched with 22,610 comparators, and 3,810 patients who underwent superselective vagotomy, who were matched with 38,090 comparators (Fig. 1). The truncal vagotomy cohort had higher Charlson comorbidity index scores than the comparison cohort; otherwise both groups were well balanced according to age, sex, and calendar year of the index date. The superselective vagotomy and comparison cohorts had similar characteristics. Notably, direct comparison of the two vagotomy cohorts indicated that patients who underwent truncal vagotomy rather than superselective vagotomy were more likely to be female, to be older, to have undergone vagotomy at a later date, and to have higher Charlson comorbidity index scores (Table 1).

**Table 1:** Characteristics of the vagotomy and matched comparison cohorts

	Truncal Vagotomy Cohort (*n* = 2260)	General Population Comparison Cohort (*n* = 22610)	Superselective Vagotomy Cohort (*n* = 3810)	General Population Comparison Cohort (*n* = 38090)
Age group, years, n* (%)				
18–40	210 (9.0)	2070 (9.2)	920 (24.0)	9170 (24.1)
41–50	380 (17.0)	3840 (17.0)	1120 (29.3)	11,170 (29.3)
51–60	500 (22.0)	5020 (22.2)	990 (26.1)	9940 (26.1)
61–70	540 (23.8)	5380 (23.8)	610 (16.0)	6110 (16.0)
71+	630 (27.8)	6300 (27.9)	170 (4.5)	1700 (4.5)
Sex, n* (%)				
Female	940 (41.4)	9420 (41.6)	1250 (32.7)	12,470 (32.7)
Male	1320 (58.2)	13,190 (58.3)	2560 (67.2)	25,620 (67.2)
Calendar year, n* (%)				
1977–1981	500 (21.8)	4960 (21.9)	1540 (40.3)	15,360 (40.3)
1982–1986	660 (29.1)	6600 (29.2)	1270 (33.3)	12,720 (33.4)
1987–1991	760 (33.7)	7640 (33.8)	730 (19.2)	7280 (19.1)
1992–1995	340 (15.0)	3410 (15.1)	270 (7.2)	2730 (7.2)
Charlson comorbidity index score, n* (%)				
0	1760 (77.5)	20,260 (89.6)	3570 (93.7)	36,450 (95.7)
1–2	410 (18.3)	2080 (9.2)	210 (5.6)	1520 (4.0)
≧ 3	90 (3.7)	270 (1.2)	30 (0.7)	120 (0.3)

*Rounded to the nearest 10

### Outcomes

**Fig. 2:**
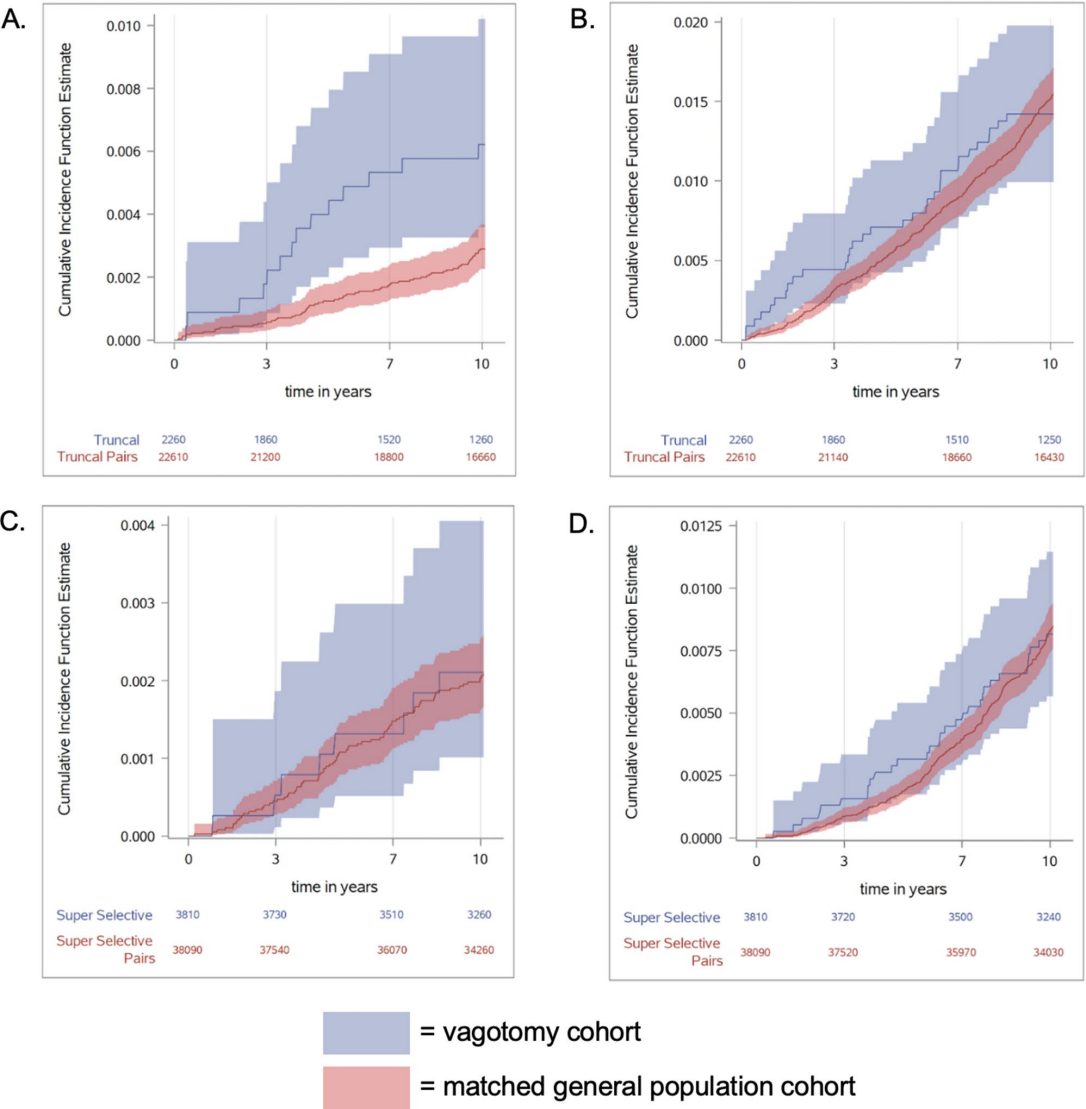
Cumulative incidence estimates for **A**. rheumatoid arthritis and **B**. osteoarthritis in patients who underwent truncal vagotomy compared with comparison subjects from the general population, and for **C**. rheumatoid arthritis and **D**. osteoarthritis in patients who underwent superselective vagotomy compared with comparison subjects from the general population. The incidence of RA and OA was calculated with a cumulative incidence function, with death treated as a competing event, and the incidence rate per 1,000 person-years reported

Incident RA was observed in 10 patients in the truncal vagotomy cohort, with an IR (95% CI) of 10.2 (6.5–15.3) per 1,000 person-years, versus 70 patients in the matched comparison cohort (IR 7.2 [6.1–8.4] per 1,000 person-years) (Table 2). Incident RA was also observed in 10 patients in the superselective vagotomy cohort, with an IR (95% CI) of 12.1 (8.8–16.1) per 1,000 person-years, versus 80 patients in the matched comparison cohort (IR 8.9 [8.0–9.9] per 1,000 person-years) (Table 2). The IR (95% CI) per 1,000 person-years for OA was 46.9 (38.4–56.7) and 70.2 (66.8–73.7) for the truncal vagotomy patients and comparison cohorts, respectively, and 85.9 (76.8–95.7) and 87.1 (84.2–90.2) for the superselective vagotomy and comparison cohorts, respectively. Cumulative incidence estimates of RA and OA in the truncal and superselective vagotomy cohorts, and corresponding comparison cohorts, are depicted in Fig. 2.

**Table 2:** Incidence of development of RA, OA, and OA with joint replacement after truncal or superselective vagotomy, with respect to matched general population comparison cohorts

	Truncal Vagotomy Cohort (*n* = 2260)	General Population Comparison Cohort (*n* = 22610)	Superselective Vagotomy Cohort (*n* = 3810)	General Population Comparison Cohort (*n* = 38090)
Incident RA, n* (%)	10 (0.7)	70 (0.3)	10 (0.3)	80 (0.2)
IR (95% CI) for RA	10.2 (6.5–15.3)	7.2 (6.1–8.4)	12.1 (8.8–16.1)	8.9 (8.0–9.9)
Crude HR (95% CI) for RA	2.49 (1.40–4.43)	1.0	1.05 (0.51–2.16)	1.0
Adjusted HR (95% CI)** for RA	2.62 (1.47–4.67)	1.0	1.05 (0.51–2.17)	1.0
Incident OA, n* (%)	30 (1.3)	350 (1.5)	30 (0.8)	340 (0.9)
IR (95% CI) for OA	46.9 (38.4–56.7)	70.2 (66.8–73.7)	85.9 (76.8–95.7)	87.1 (84.2–90.2)
Crude HR (95% CI) for OA	1.11 (0.77–1.59)	1.0	0.98 (0.68–1.40)	1.0
Adjusted HR (95% CI)** for OA	1.26 (0.88–1.81)	1.0	1.00 (0.69–1.44)	1.0
Incident OA w/JR, n* (%)	10 (0.4)	160 (0.7)	10 (0.3)	120 (0.3)
IR (95% CI) for OA w/JR	25.7 (19.5–33.2)	45.7 (42.9–48.6)	50.4 (43.5–58.1)	60.4 (57.9–62.9)
Crude HR (95% CI) for OA w/JR	0.61 (0.30–1.23)	1.0	0.69 (0.34–1.40)	1.0
Adjusted HR (95% CI)** for OA w/JR	0.77 (0.38–1.57)	1.0	0.71 (0.35–1.46)	1.0

RA = rheumatoid arthritis; OA = osteoarthritis; IR = incidence rate per 1,000 person-years; HR = hazard ratio; 95% CI = 95% confidence interval; JR = joint replacement. *Rounded to the nearest 10. **Adjusted for age, sex, and calendar year

After adjustment for age, sex, and calendar year, the aHR (95% CI) for RA development was 2.62 (1.47–4.67) in the truncal vagotomy cohort and 1.05 (0.51–2.17) in the superselective vagotomy cohort, with respect to the matched comparison cohorts (Table 2). The aHR (95% CI) for OA development was 1.26 (0.88–1.81) in the truncal vagotomy cohort and 1.00 (0.69–1.42) in the superselective vagotomy cohort, with respect to the matched comparison cohorts (Table 2). Similar results were obtained for OA with joint replacement. The aHR (95% CI) for RA development was 3.08 (1.22–7.79) in the truncal vagotomy cohort, and the aHR (95% CI) for OA development was 1.12 (0.64–1.94), with respect to the superselective vagotomy cohort (Table 3). Covariate estimates from the multivariable Cox proportional hazards regression models are presented in Supplementary Tables 5 and 6.

**Table 3:** Incidence of development of RA, OA, and OA with joint replacement after truncal vagotomy versus superselective vagotomy

	Truncal Vagotomy Cohort (*n* = 2260)	Superselective Vagotomy Cohort (*n* = 3810)
Incident RA, n* (%)	10 (0.7)	10 (0.3)
IR (95% CI) for RA	10.2 (6.5–15.3)	12.1 (8.8–16.1)
Crude HR (95% CI) for RA	3.68 (1.54–8.78)	1.0
Adjusted HR (95% CI)* for RA	3.08 (1.22–7.79)	1.0
Incident OA, n* (%)	30 (1.3)	30 (0.8)
IR (95% CI) for OA	46.9 (38.4–56.7)	85.9 (76.8–95.7)
Crude HR (95% CI) for OA	2.16 (1.32–3.53)	1.0
Adjusted HR (95% CI)* for OA	1.12 (0.64–1.94)	1.0
Incident OA w/JR, n* (%)	10 (0.4)	10 (0.3)
IR (95% CI) for OA w/JR	25.7 (19.5–33.2)	50.4 (43.5–58.1)
Crude HR (95% CI) for OA w/JR	2.24 (0.84–5.97)	1.0
Adjusted HR (95% CI)* for OA w/JR	0.61 (0.19–1.90)	1.0

RA = rheumatoid arthritis; OA = osteoarthritis; IR = incidence rate per 1,000 person-years; HR = hazard ratio; 95% CI = 95% confidence interval; JR = joint replacement. *Rounded to the nearest 10. **Adjusted for age, sex, and calendar year

### Sensitivity analysis

Given the strong competing risk of death in the truncal vagotomy cohort, we used the Fine-Gray model, which yielded similar results to the Cox proportional hazards regression models (Supplementary Tables 7 and 8).

## Discussion

We used longitudinal Danish health registry data to study the effects of vagotomy on RA development. Patients who underwent full truncal vagotomy, which completely interrupts vagus nerve signaling to the spleen, had a more than 2.5-fold greater risk of RA development than people from the general population. This effect was not observed for superselective vagotomy, which only interrupts the vagal nerve fibers that directly innervate the stomach, thereby preserving vagus nerve signaling to the spleen. Furthermore, neither vagotomy method was associated with elevated risk of OA. Unlike RA, which is driven by an overactive and misdirected immune response, OA is a non-inflammatory form of arthritis. Thus, it is not expected that alterations in the inflammatory reflex would affect OA development. Finally, patients who underwent truncal vagotomy had a 3-fold greater risk of RA development than those who underwent superselective vagotomy. Current clinical trials are assessing the effects of vagus nerve stimulation on RA disease activity. Herein, we took a different approach to the question by evaluating the risk of RA development associated with disruption of vagus nerve signaling by vagotomy. Although our results provide indirect evidence, they support the theoretical basis for vagus nerve stimulation for RA treatment.

Vagus nerve stimulation is believed to function through the inflammatory reflex, which consists of an afferent sensory arm that detects inflammatory molecules, and an efferent motor neural arm that transmits signals modulating immune responses [8]. Vagus nerve stimulation typically has anti-inflammatory effects by triggering acetylcholine release in the spleen, thus inhibiting proinflammatory cytokine production by macrophages [4–8]. These cytokines, such as tumor necrosis factor-alpha, are known to play critical roles in RA pathogenesis [1]. Thus, we hypothesized that disruption of vagus nerve connections (e.g. via full truncal vagotomy) worsens inflammation and increases the risk of developing inflammatory disease such as RA. This has been demonstrated in a mouse model of inflammatory arthritis, in which vagotomy exacerbated disease, however it remains unknown whether vagus nerve disruption has a similar effect in humans [17]. To date, studies of the vagus nerve pathway in RA patients have primarily centered around vagus nerve stimulation, with mixed results. Several such studies have used a cervically implanted VNS device. The first open-label trial in 17 patients with RA demonstrated a decrease in disease activity score of 28 joints with C-reactive protein (DAS28-CRP) by 1.89 at day 42 [5]. In a second study, 11 patients with active RA and prior inadequate response to two or more biologic disease-modifying antirheumatic drugs (bDMARDs) or targeted synthetic disease-modifying antirheumatic drugs (tsDMARDs) were treated for 12 weeks with one minute of VNS once daily, one minute of VNS four times daily, or no stimulation. The patients receiving stimulation four times daily had DAS28-CRP decrease of 1.2, whereas no improvement was observed in those receiving once daily stimulation or no stimulation [9]. In addition, non-invasive, auricular VNS has been evaluated for RA treatment. In an open-label study in 30 patients with RA with inadequate response to csDMARDs and up to one bDMARD, auricular VNS decreased DAS28-CRP by 1.4 at week 12, and 16 (53%) patients achieved 20% improvement in American College of Rheumatology criteria (ACR20) [18]. However, in a subsequent randomized, double-blind, sham-controlled study, 12 weeks of treatment with auricular VNS, compared with sham stimulation, did not improve the ACR response criteria or DAS28-CRP [10].

A prior case-control study using the Swedish Inpatient Register assessed vagotomy and RA risk in humans [19]. In that study, among 63,209 patients with prevalent RA, 179 patients had previously undergone vagotomy, whereas 304 patients had undergone vagotomy among the 125,404 matched general population controls. The odds ratio (OR) (95% CI) was 1.17 (0.97–1.40) for the association between vagotomy history and RA. For incident RA, 10 of 2,458 patients with RA had undergone vagotomy, compared with 64 of 24,357 controls (OR 1.56 [0.65–3.76]). The study concluded that vagotomy was not associated with elevated risk of RA development. Our contrasting results might be ascribed to differences in design (population-based cohort study with prospectively collected data versus a case-control study), and the covariates used in matching. In addition, the prior study did not distinguish between truncal and selective forms of vagotomy. By combining all forms of vagotomy in the analysis, the association between truncal vagotomy and RA was likely to have been lost.

Our study has several strengths. We used a large, high quality, nationwide database encompassing more than 8 million individuals in Denmark. We assessed disruption of vagal nerve activity, which would be impractical to investigate in an interventional trial. In addition, only one prior study has epidemiologically evaluated the association between vagotomy and the risk of subsequent RA development; moreover, our study is the first to assess the risk of OA development.

Our study also has several limitations. First, this was a registry-based study, and the use of ICD and procedure codes can lead to misclassification of both exposures and outcomes. The sensitivity and specificity of RA and OA diagnosis codes in the DNPR have not been evaluated, and thus misclassification bias cannot be ruled out. We used two or more ICD codes separated by 7 to 365 days to determine the outcomes to increase the likelihood of identifying true cases. However, this algorithm has a relatively low specificity and positive predictive value [15, 20, 21]. We were unable to access medication history to improve the accuracy of RA detection. To some degree, this misclassification might not have differed between groups; however, patients who underwent vagotomy might potentially have fundamentally differed from those who did not, and the basic covariates used for matching (age, sex, and calendar year) were probably inadequate for eliminating residual confounding. This potential misclassification of the outcome may have resulted in bias in either direction and significantly impacted the results given the small total number of RA cases that were identified. Second, given the higher Charlson comorbidity scores in patients who underwent truncal vagotomy than in matched controls, those patients might potentially have accrued more diagnosis codes as a function of having more health system interactions, thus potentially resulting in observation of more diagnoses of RA. If this were the case, we would have expected similar results for OA development risk in patients who underwent truncal vagotomy, which was not observed. Third, we were unable to include variables such as medication use, smoking, which is associated with RA, or body mass index, which is associated with OA. If these variables are associated with one exposure more than the other, the results may be confounded. For instance, if smoking is more common in patients who underwent full truncal vagotomy, and smoking is also associated with the development of RA, the results could be biased in favor of an association between full truncal vagotomy and the development of RA. In future studies, these variables should be adjusted for. Finally, the competing risk of death was significant in the truncal vagotomy cohort. However, we performed a sensitivity analysis using the Fine-Gray model, which models the subdistribution hazard attached to the cumulative incidence and can be interpreted as the instantaneous risk of experiencing the event of interest in the presence of competing risks, which yielded similar results to the primary analysis [22].

## Conclusions

The aim of this study was to evaluate the relation between vagotomy and RA risk. We hypothesized that patients who had undergone full truncal vagotomy would have greater risk of RA development than matched comparison cohort members and patients who had undergone superselective vagotomy, thus retaining an intact inflammatory reflex. Indeed, truncal vagotomy was associated with elevated RA incidence. This supports the hypothesis that disruption of vagus nerve signaling might influence the inflammatory reflex and contribute to RA development. Our work highlights the importance of ongoing and future prospective, interventional studies of VNS in patients with RA, which will provide more definitive evidence of the role of the vagus nerve in RA.

## Electronic supplementary material

Below is the link to the electronic supplementary material.

Supplementary Material 1

## Data Availability

No datasets were generated or analysed during the current study.

## Abbreviations

ACR: American College of Rheumatology
bDMARD: Biologic Disease-Modifying Antirheumatic Drug
CI: Confidence Interval
CRS: Danish Civil Registration System
DAS28-CRP: Disease Activity Score of 28 Joints with C-Reactive Protein
DMARD: Disease-Modifying Antirheumatic Drug
DNPR: Danish National Patient Registry
HR: Hazard Ratio
ICD: International Classification of Diseases
IR: Incidence Rate
OA: Osteoarthritis
OR: Odds Ratio
PUD: Peptic Ulcer Disease
RA: Rheumatoid Arthritis
tsDMARD: Targeted Synthetic Disease-Modifying Antirheumatic Drug
VNS: Vagus Nerve Stimulation
