# Supplementary material for: Vagotomy and the incidence of rheumatoid arthritis and osteoarthritis: a Danish register-based study
Source: Arthritis Res Ther. 2025 May 16;27:106. doi: 10.1186/s13075-025-03567-y (PMC12083144; doi:10.1186/s13075-025-03567-y)
Supplement: Supplementary file 1 — Supplementary Material 1 [file 13075_2025_3567_MOESM1_ESM.docx]

**Supplemental Online Content**

**Vagotomy and the Incidence of Rheumatoid Arthritis and Osteoarthritis: A Danish Register-Based Study**

Matthew C. Baker MD MS, Dávid Nagy MSc, Suzanne Tamang PhD MS, Erzsébet Horváth-Puhó MSc PhD, Henrik Toft Sørensen MD PhD DMSc Dsc

**Supplementary Table 1.** Danish Classification of Surgical Procedure and Therapies Codes used to identify vagotomy.

**Supplementary Table 2.** ICD codes used to identify OA and RA.

**Supplementary Table 3.** Procedure codes used for OA with joint replacement outcome.

**Supplementary Table 4.** ICD codes used to calculate the Charlson comorbidity index.

**Supplementary Table 5.** Adjusted hazard ratios of exposure and covariables from multivariable Cox proportional hazards regression models when comparing truncal vagotomy cohorts with matched general population comparison cohorts.

**Supplementary Table 6.** Adjusted hazard ratios of exposure and covariables from multivariable Cox proportional hazards regression models when comparing superselective vagotomy cohorts with matched general population comparison cohorts.

**Supplementary Table 7.** Incidence of development of RA, OA, and OA with joint replacement after truncal or superselective vagotomy, with respect to matched general population comparison cohorts, using Fine-Gray.

**Supplementary Table 8.** Incidence of development of RA, OA, and OA with joint replacement after truncal vagotomy versus superselective vagotomy using Fine-Gray.

| **Supplementary Table 1.** Danish Classification of Surgical Procedure and Therapies Codes used to identify vagotomy. | |
| --- | --- |
| **Truncal** | |
| **Operation Code** | **Diagnosis** |
| 34310 | TRANSTHORACALIS VAGOTOMY |
| 34820 | VAGOTOMIA TRUNCALIS THORACALIS |
| 42620 | VAGOTOMIA TRUNCALIS ABDOMINALIS |
| 42621 | VAGOTOMIA TRUNCALIS ABDOMINALIS LAPAROSCOPICA |
| **Selective** | |
| **Operation Code** | **Diagnosis** |
| 42680 | VAGOTOMIA SELECTIVA VENTRICULI |
| 42681 | VAGOTOMIA SELECTIVA VENTRICULI LAPAROSCOPICA |
| **Superselective** | |
| **Operation Code** | **Diagnosis** |
| 42720 | VAGOTOMIA AREAE ACIDOGENIS VENTRICULI |
| 42742 | VAGOTOMIA AREAE ACIDOGENIS VENTRICULI ENDOSCOPICA |

| **Supplementary Table 2.** ICD codes used to identify OA and RA. | |
| --- | --- |
| **ICD Codes for Osteoarthritis** | |
| **ICD-8** | **Diagnosis** |
| 713.00, 713.01, 713.02, 713.03, 713.04, 713.05, 713.06, 713.08, 713.09 | Osteoarthritis |
| **ICD-10** | **Diagnosis** |
| M15, M15.0, M15.1, M15.2, M15.3, M15.3A, M15.4, M15.8, M15.9 | Polyosteoarthritis |
| M16. M16.0, M16.1, M16.1A, M16.2, M16.3, M16.3A, M16.4, M16.5, M16.5A, M16.6, M16.7, M16.7A, M16.9 | Osteoarthritis of hip |
| M17, M17.0, M17.1, M17.2, M17.3, M17.4, M17.5, M17.9 | Osteoarthritis of knee |
| M18, M18.0, M18.1, M18.1A, M18.2, M18.3, M18.3A, M18.4, M18.5, M18.5A, M18.9 | Osteoarthritis of first carpometacarpal joint |
| M19, M19.0, M19.0A M19.1, M19.1A M19.2, M19.2A M19.8, M19.8A, M19.9 | Other and unspecified osteoarthritis |
| **ICD Codes for Rheumatoid Arthritis** | |
| **ICD-8** | **Diagnosis** |
| 712.19, 712.29, 712.39, 712.59 | Rheumatoid arthritis and allied conditions |
| **ICD-10** | **Diagnosis** |
| M05, M05.0, M05.1, M05.1A, M05.1B, M05.1C, M05.1D, M05.1E, M05.1F, M05.2, M05.3, M05.8, M05.9, M06, M06.2, M06.3, M06.4, M06.8, M06.9 | Rheumatoid arthritis |
| ICD = international classification of diseases. | |

| **Supplementary Table 3.** Procedure codes used for OA with joint replacement outcome. | |
| --- | --- |
| **CPT Codes for Arthroplasty** | |
| **CPT Code** | **Diagnosis** |
| 27120, 27122, 27125, 27130 | Hip arthroplasty |
| 27438, 27440, 27441, 27442, 27443, 27445, 27446, 27447 | Knee arthroplasty |
| **NOMESCO with K Prefix Codes** | **Diagnosis** |
| KNFB, KFNC | Hip arthroplasty |
| KNGB, KNGC | Knee arthroplasty |
| **Danish Classification of Surgical Procedure and Therapies Codes** | **Diagnosis** |
| 70030, 70031, 70032, 70033, 70034, 70035, 70036, 70039 | Hip arthroplasty |
| 70040, 70041, 70042, 70043, 70044, 70045, 70046, 70049 | Knee arthroplasty |
| **ICD-8 Codes** | **Diagnosis** |
| 7003, 8274 | Hip arthroplasty |
| 7004, 8278 | Knee arthroplasty |
| CPT = current procedural terminology. | |

| **Supplementary Table 4.** ICD codes used to calculate the Charlson comorbidity index. | |
| --- | --- |
|  | |
| **ICD-8** | **Diagnosis** |
| 410 | Myocardial infarction |
| 427.09; 427.10; 427.11; 427.19; 428.99; 782.49 | Congestive heart failure |
| 440–445 | Peripheral vascular disease |
| 430–438 | Cerebrovascular disease |
| 490-493; 515-518 | Chronic pulmonary disease |
| 249.00; 249.06; 249.07; 249.09; 250.00; 250.06; 250.07; 250.09 | Diabetes without chronic complication |
| 249.01–249.05; 249.08; 250.01–250.05; 250.08 | Diabetes with chronic complication |
| **ICD-10** | **Diagnosis** |
| I21.x, I22.x, I25.2 | Myocardial infarction |
| I09.9, I11.0, I13.0, I13.2, I25.5, I42.0, I42.5 - I42.9, I43.x, I50.x, P29.0 | Congestive heart failure |
| I70.x, I71.x, I73.1, I73.8, I73.9, I77.1, I79.0, I79.2, K55.1, K55.8, K55.9, Z95.8, Z95.9 | Peripheral vascular disease |
| G45.x, G46.x, H34.0, I60.x - I69.x | Cerebrovascular disease |
| I27.8, I27.9, J40.x - J47.x, J60.x - J67.x, J68.4, J70.1, J70.3 | Chronic pulmonary disease |
| E10.0, E10.1, E10.6, E10.8, E10.9, E11.0, E11.1, E11.6, E11.8, E11.9, E12.0, E12.1, E12.6, E12.8, E12.9, E13.0, E13.1, E13.6, E13.8, E13.9, E14.0, E14.1, E14.6, E14.8, E14.9 | Diabetes without chronic complication |
| E10.2 - E10.5, E10.7, E11.2 - E11.5, E11.7, E12.2 - E12.5, E12.7, E13.2 - E13.5, E13.7, E14.2 - E14.5, E14.7 | Diabetes with chronic complication |
| ICD = international classification of diseases. | |

| **Supplementary Table 5.** Adjusted hazard ratios and covariate estimates from multivariable Cox proportional hazards regression models when comparing truncal vagotomy cohorts with matched general population comparison cohorts. | | | |
| --- | --- | --- | --- |
|  | **Adjusted HR* for RA:  Truncal Vagotomy Cohort  vs Comparison Cohort** | **Adjusted HR for OA:  Truncal Vagotomy Cohort  vs Comparison Cohort** | **Adjusted HR for OA w/JR:  Truncal Vagotomy Cohort  vs Comparison Cohort** |
| Exposure | 2.62 (1.47-4.67) | 1.26 (0.88-1.81) | 0.77 (0.38-1.57) |
| Male sex | REF | REF | REF |
| Female sex | 1.78 (1.14-2.78) | 1.50 (1.22-1.84) | 1.54 (1.13-2.11) |
| Age < 41 | REF | REF | REF |
| Age 41-51 | 3.13 (0.70-14.01) | 2.64 (1.10-6.31) | 4.93 (0.64-38.20) |
| Age 51-61 | 2.99 (0.68-13.18) | 5.00 (2.18-11.49) | 12.11 (1.66-88.60) |
| Age 61-71 | 7.19 (1.72-30.00) | 7.90 (3.48-17.97) | 22.60 (3.13-163.16) |
| Age ≥ 71 | 3.94 (0.91-17.04) | 10.15 (4.48-23.02) | 23.89 (3.31-172.44) |
| Period 1977-1981 | REF | REF | REF |
| Period 1982-1986 | 1.73 (0.89-3.38) | 1.40 (0.96-2.04) | 5.95 (1.35-26.21) |
| Period 1987-1991 | 1.59 (0.82-3.10) | 2.87 (2.05-4.02) | 33.48 (8.24-136.02) |
| Period 1992-1995 | 2.03 (0.96-4.30) | 3.44 (2.39-4.95) | 50.83 (12.41-208.23) |
| RA = rheumatoid arthritis; OA = osteoarthritis; HR = hazard ratio; 95% CI = 95% confidence interval; JR = joint replacement. *Adjusted for age, sex, and calendar year. | | | |

| **Supplementary Table 6.** Adjusted hazard ratios and covariate estimates from multivariable Cox proportional hazards regression models when comparing superselective vagotomy cohorts with matched general population comparison cohorts. | | | |
| --- | --- | --- | --- |
|  | **Adjusted HR* for RA:  Superselective Vagotomy Cohort  vs Comparison Cohort** | **Adjusted HR for OA:  Superselective Vagotomy Cohort  vs Comparison Cohort** | **Adjusted HR for OA w/JR:  Superselective Vagotomy Cohort  vs Comparison Cohort** |
| Exposure | 1.05 (0.51-2.17) | 1.00 (0.69-1.44) | 0.71 (0.35-1.46) |
| Male sex | REF | REF | REF |
| Female sex | 3.22 (2.07-5.00) | 1.39 (1.13-1.71) | 1.35 (0.95-1.91) |
| Age < 41 | REF | REF | REF |
| Age 41-51 | 4.77 (1.42-16.06) | 2.48 (1.64-3.76) | 5.03 (2.11-12.00) |
| Age 51-61 | 9.14 (2.80-29.80) | 3.48 (2.30-5.25) | 7.47 (3.13-17.79) |
| Age 61-71 | 10.77 (3.24-35.80) | 7.63 (5.10-11.44) | 15.85 (6.70-37.48) |
| Age ≥ 71 | 4.88 (0.98-24.26) | 8.84 (5.38-14.51) | 15.45 (5.58-42.80) |
| Period 1977-1981 | REF | REF | REF |
| Period 1982-1986 | 0.92 (0.57-1.48) | 1.30 (0.99-1.71) | 3.96 (2.10-7.45) |
| Period 1987-1991 | 0.82 (0.44-1.53) | 2.78 (2.12-3.65) | 10.37 (5.59-19.21) |
| Period 1992-1995 | 0.72 (0.26-2.00) | 4.04 (2.92-5.58) | 18.38 (9.59-35.20) |
| RA = rheumatoid arthritis; OA = osteoarthritis; HR = hazard ratio; 95% CI = 95% confidence interval; JR = joint replacement. *Adjusted for age, sex, and calendar year. | | | |

| **Supplementary Table 7.** Incidence of development of RA, OA, and OA with joint replacement after truncal or superselective vagotomy, with respect to matched general population comparison cohorts, using Fine-Gray. | | | | |
| --- | --- | --- | --- | --- |
|  | **Truncal Vagotomy Cohort**  **(*n* = 2260)** | **General Population Comparison Cohort**  **(*n* = 22610)** | **Superselective Vagotomy Cohort (*n* = 3810)** | **General Population Comparison Cohort  (*n* = 38090)** |
| Adjusted HR (95% CI)* for RA | 2.16 (1.21–3.85) | 1.0 | 1.04 (0.50–2.15) | 1.0 |
| Adjusted HR (95% CI)* for OA | 0.94 (0.65–1.35) | 1.0 | 0.99 (0.68–1.43) | 1.0 |
| Adjusted HR (95% CI)* for OA w/JR | 0.51 (0.25–1.05) | 1.0 | 0.68 (0.31–1.45) | 1.0 |
| RA = rheumatoid arthritis; OA = osteoarthritis; HR = hazard ratio; 95% CI = 95% confidence interval; JR = joint replacement. *Adjusted for age, sex, and calendar year. | | | | |

| **Supplementary Table 8.** Incidence of development of RA, OA, and OA with joint replacement after truncal vagotomy versus superselective vagotomy using Fine-Gray. | | |
| --- | --- | --- |
|  | **Truncal Vagotomy  Cohort**  **(*n* = 2260)** | **Superselective Vagotomy Cohort** **(*n* = 3810)** |
| Adjusted HR (95% CI)* for RA | 2.89 (1.20–6.96) | 1.0 |
| Adjusted HR (95% CI)* for OA | 0.97 (0.52–1.81) | 1.0 |
| Adjusted HR (95% CI)* for OA w/JR | 0.45 (0.16–1.23) | 1.0 |
| RA = rheumatoid arthritis; OA = osteoarthritis; HR = hazard ratio; 95% CI = 95% confidence interval; JR = joint replacement. *Adjusted for age, sex, and calendar year. | | |
